# Book Reviews

**Published:** 1820-10

**Authors:** 


### CRITICAL ANALYSIS

OF

### RECENT PUBLICATIONS, IN THE DIFFERENT BRANCHES OF MEDICINE AND SURGERY.

"I would have men know, that though I reprehend the easie passing over of the causes of things, " by ascribing them to secret and hidden vertues and properties; (for this hath arrested and laid " asleepe all true enquiry and indications;) yet I doe not understand but that, in the practical " part of knowledge, much will be left to experience and probation, whereunto indication cannot " so fully reach: and this not only in *specie*, but in *individuo*. Yet it was well said, *Vere scire* " *esse per causas scire*."—BACON.

*A Treatise on the Diseases of the Urethra, Vesica Urinaria, Prostate, and Rectum.* By CHARLES BELL, Surgeon to the Middlesex Hospital; and Lecturer on Anatomy in the School of Great Windmill-street. A new Edition. With Notes, containing the Criticisms of the Editors of the Foreign Editions, and the Opinions of Foreign Authors on these Diseases. By JOHN SHAW, Surgeon; Demonstrator of Anatomy in the School of Great Windmill-street. 8vo. pp. 416. Longman and Co. 1820.

THE dissertations which this volume contains, (the author states in a Preface,) were written at intervals, and have received his occasional corrections during fifteen years. "When a case was attended with unusual anxiety, or when a dissection was made, or a preparation was brought to him, which illustrated something of this part of pathology, the author has been in the habit of adding to his notes. It is on the information thus zealously and cautiously collected, that he rests his hopes of the approbation of the profession." Those who have perused the work will not, we believe, doubt but that those hopes will be realized to their fullest extent, and accompanied with feelings of gratitude of no ordinary kind for the benefits the profession must derive from these results of his clinical experience and anatomical researches; for, without information of this kind, those best acquainted with the general principles of pathology and surgery find themselves perplexed, if not absolutely at a loss for proper measures, in the treatment of the more serious cases of the diseases which are the objects of these dissertations, more than in that of any other class of maladies. Although the author has here produced what may be considered a general treatise on the diseases of the urethra, his especial object appears to have been to describe the nature and consequences of the cases just alluded to, as well as to show what should be the conduct of the surgeon in the management of them; and we shall not, we think, assert what is not strictly correct, if we say that his means for effecting these intentions have been *singularly* advantageous.

The contributions of his annotator, designated in the title-page, add considerably to the practical value of the work, as we shall have occasion to point out in the course of the analysis we are about to enter on; and a preliminary "description of the anatomy of some parts of the urethra and neck of the bladder," is also the production of Mr. Shaw. This description may be passed over here, as we inserted his observations on this subject in the last Number of this Journal.

Mr. Bell, in the first chapter, adduces some original notions respecting the sensibility of the bladder, and some of its morbid affections, which are highly interesting: they are qualified to elucidate some important things in pathology, and suggest the means for relieving at least one troublesome affection, which has hitherto been rebellious to all our measures. These are preluded by some observations on the situation and arrangement of the sphincter of the bladder, which we shall here adduce in detail.

"If we consider the double office of the urethra, and suppose that the seminal vessels, and the ducts of the prostate gland, open into the canal at a part posterior to the muscles which close the orifice of the bladder, we must be also forced to admit that there is some imperfection in the mechanism of these parts. For, in that case, the fluids passing from those ducts would fall back into the bladder, and the orifices of the ducts would be exposed to the urine in the bladder, even when the bladder was closed. If this were really the case, it would be inconceivable how the contents of the *vesiculæ seminales* could be discharged forwards, or how the urine could be retained while the seminal discharge was made.

"By such a train of reasoning, I was led to look for the proper sphincter of the bladder behind the prostate. The importance of the knowledge of the complex apparatus of muscles about the neck of the bladder, to the comprehension of the various causes of obstructed urine, led me to review this part of the anatomy.

"To exhibit the sphincter of the bladder, cut off all the appendages but the prostate gland; then make an incision into the fundus of the bladder, and invert it. Begin the dissection, by taking off the inner membrane of the bladder from around the orifice of the urethra. A set of fibres will be discovered on the lower half of the orifice, which, being carefully dissected, will be found to run in a semicircular form round the urethra. These fibres make a band of about half an inch in breadth, particularly strong on the lower part of the opening, and, having mounted a little above the orifice on each side, they disperse a portion of their fibres in the substance of the bladder. A smaller and somewhat weaker set of fibres will be seen to complete their course, surrounding the orifice on the upper part: to these sphincter fibres a bridle is joined, which comes from the union of the muscles of the ureters. Here, then, we have the muscle which closes the internal extremity of the urethra, the most posterior of all those muscles which embrace the urethra. It resembles the sphincters of the other hollow viscera: for example, those fibres which encircle the pyloric orifice of the stomach."

All anatomists since GALEN seem to have admitted the existence of a proper sphincter of the bladder at its neck, with the exception of Bianchi, Palucci, and Lieutaud, who pretended that what was regarded as a proper sphincter was only the termination of the fibres of the muscular coat of the bladder, the *detrusor urinæ*: but the precise arrangement of this structure does not appear to have been understood before these researches of Mr. Bell, which may probably be considered finally decisive of the disputed point just alluded to.

Directly over the junction of the muscles of the ureters with the internal sphincter of the bladder, the internal coat of the bladder, the author says, is found to be more vascular than at any other part. This is the spot of the bladder which is beyond all comparison the most sensitive; and, however painful the operation of the catheter may be, the patient does not suffer the call to make urine while it is passing along the urethra, nor until it touches this part; but, as soon as the catheter, or bougie, rubs upon this sensitive spot, he says "he must make water; he cannot retain the water longer;" and he feels the call, although there be not a drop of urine in the bladder. When a calculus rests here, the patient suffers what he calls a fit of the stone; for, while it touches this part, there is an unceasing *nisus;* the bladder is stimulated to contraction, and the coats come in contact with the surface of the stone. SAMUEL SHARP, as the author shows, was aware of this circumstance; but he supposed that it might be accounted for from the stone "touching the bladder in more points when it lies in the neck, than when it is in its body or fundus; in consequence of which it must irritate more." Mr. Bell argues that this explanation is not satisfactory; for, he says, "it is not an increase of pain merely which the patient experiences when the stone falls forward from its common place of lodgment; it is the call to make water; it is the pain of retained urine, and the sudden spasmodic action of the bladder, excited by the presence of the stone on this *sensible spot*, attended with a squirting of the urine as from the bladder of a dog."

This sensibility, the author adds, is neither accidental nor morbid. It proceeds from a nerve distributed here, and which has its branches ramifying to all the muscles engaged in the act of passing urine. The immediate effect of the exercise of this sensibility, is the action of the muscles of the ureters, and the relaxation of the sphincter; but, by the connection of nerves, there is, at the same time, a consent widely established among the numerous muscles engaged in the act of discharging the contents of the bladder. The proper internal sphincter of the bladder,—the compressor prostatæ,—the levator ani,—the levator or compressor urethræ of Mr. Wilson,—the ejaculator seminis,—and the internal and oblique perinæal muscles, are, Mr. Bell says, all of one class: they must relax by consent with the sphincter, else the urine cannot flow. They may be said to be of the class of sphincter muscles. Their opponents are the detrusor urinæ or muscular coat of the bladder, and (in consent with it) the abdominal muscles and diaphragm. Mr. Bell illustrates the various connected and related actions of these two classes of muscles, by stating the effects of irritation of the nostrils, glottis, and pharynx, on the muscles of respiration.

The ideas of Mr. Bell respecting this peculiarly sensible spot of the bladder, serve to explain also, in a satisfactory manner, the cause of incontinence of urine during sleep, which is often so distressing an affection in children. This incontinence, the author says, never takes place but when the boy is asleep upon his back. "The circumstance is unaccountable, until we reflect on the position of this master-spring of the muscles of the bladder, the sensible spot a little behind and below the orifice of the bladder. When a person lies upon his belly, the urine gravitates towards the fundus; but, when he lies on his back, it presses upon this sensible spot, and distends that part of the bladder which is towards the rectum." When a child passes its urine during sleep, it is in consequence of a dream, excited by the irritation of this sensible spot of the bladder. The cure is simple; it is merely necessary to accustom the child to sleep upon his side, with the cheek on the pillow: the urine is then not passed, nor is the patient excited to dream of making urine while he keeps this position.

An increase of the sensibility of this spot of the bladder, from various causes, will also induce an incontinence of urine, troubling the boy during the day; and Mr. Shaw adds, that this may occur without the patient suffering much irritation. "In such a case," he says, "the boy may be obliged to retain his water longer, by compressing the urethra; by a careful distension of the bladder, and by gradually increasing the interval of making water, the bladder may be at last brought to contain its natural quantity."

A morbid increase of the sensibility of the bladder in the part just mentioned, will induce abscess around the prostate and vesiculæ seminales; just as long-continued inflammation of the membrane of the larynx, or of the mucous membrane of the fauces, will produce suppuration about the cartilages of the larynx, and in the duplicature of the arches of the throat.

This abscess about the process is, according to the author, a very severe and destructive affection. It is marked by frequent and painful calls to make urine, a burning sensation and violent spasms after the urine is discharged, pain in the glans penis, purulent discharge from the urethra in abundance, but not constantly. The patient is subject to cold shivering and fever; and he is pale, harassed, and wasted. On introducing a bougie, there is constant pain as it enters the neck of the bladder, and it comes out smeared with matter, and perhaps blood also. On examination by the anus, a thickening is felt around the prostate gland or vesiculæ seminales, and pressure in this situation is productive of pain.

"The causes of this complaint are violent inflammations in the urethra, aggravated by free living and debauchery, and by irritating injections, the unskilful use of bougies, caustic, and cantharides; but most of all I fear it in a scrofulous constitution."

The treatment advised by the author is on the principle of diminishing inflammation; and the means are, laxatives, the application of leeches to the verge of the anus; emollient and anodyne clysters; mucilaginous drinks; the alkalies; and opiates. In some cases, the introduction of a mercurial cerate, by the patient's finger, which is to be rubbed upon the anterior part of the rectum, has been advantageous; but all these are of less consequence than injection of the bladder; a measure of which Mr. Bell seems to think very favourably in various states of irritation of the bladder, and to which we are ourselves not less partial. In many cases of stricture, too, when the patient cannot otherwise pass a drop of water without the introduction of a bougie, it is sufficient, Mr. Bell says, to throw a little tepid water into the urethra. The presence of the injection brings on the consent of parts, and is followed by discharge of urine. During the fit of stone, by injecting tepid water, the bladder may be distended, and the stone removed from the sensible spot of the neck of the bladder. If two or three ounces of fluid be very slowly injected, the pain will be immediately mitigated.

"But," the author adds, "it is in the case of inflammation within the neck of the bladder that this injection is of the most essential service; and I much wonder that the practice has obtained so little."

He states the results of its use in five cases:

"Two of these were old gentlemen, who had symptoms of stone, and who had been repeatedly sounded, without detecting the stone; the bladder was regularly injected, and their pains were sensibly relieved, but not permanently: however, in both these cases, after using the injection, the stone was detected by sounding, and they afterwards submitted to the operation of lithotomy. Two of these patients were boys, who suffered cruelly with all the symptoms of stone. The first of these was a dwarfish boy, who was brought into the hospital with the suspicion of stone: he was sounded twice, and no stone discovered. Some months after he returned with the same complaint; a painful and frequent call to make water, with pain in the extremity of the penis. He was put under the charge of a dresser, to have the bladder injected. He expressed himself relieved from the first; gradually more and more water was admitted into the bladder; every day the bladder could contain an increased quantity of water; [urine?] and, after some weeks, he was dismissed well."

Mr. Bell, his annotator says, has found so much benefit from the injection of warm water alone, that he has been unwilling to risk the deposition of any solid matter from a compound injection. We are not aware that any ill effects have ensued from the use of injections of mucilaginous fluids, decoction of poppies, &c.; and these are certainly more beneficial in some cases than simple water. Above all, the author recommends the injection of the bladder in the commencement of the disease called uvula vesicæ. When there is an inordinate irritation of the sensible spot at the neck of the bladder, nothing is so likely to allay irritation. Mr. Bell attributes the good effects of the injection in this case to the temporary substitution of the tepid water for the urine, (which is an acrid stimulant to the bladder when in this morbid state,) and the subsequent dilution of the urine which collects in the bladder. It may also be useful, he says, by washing away the ropy mucus and purulent secretion, where there is any, by which that sort of tenesmus vesicæ caused by their presence is relieved.

The second chapter treats of the cases "which require the urine to be drawn off by instruments; and of the operation of the catheter." The greater part of this is not new to well-informed surgeons, and what is original cannot be stated in a distinct manner, so as to be of much utility. This chapter, however, though not so interesting, because not so peculiar to the author, as many others in the work, is by no means devoid of important matter; and several good cases and remarks are adduced by Mr. Shaw, in illustration of the several affections discussed by Mr. Bell.

The next chapter is on "pain and irritation felt in the bladder, urethra, and perinæum, not really seated there, but proceeding from disorder of the bowels." Affections of this kind, the author says, "bring full one-half of those patients to the surgeon who are considered as labouring under stricture of the urethra. How this subject should make no part of those numerous treatises on Stricture we possess, is very remarkable; unless we are to suppose that experience follows, and does not precede, those publications." Our worst apprehensions from pain and spasm in the bladder and urethra, he adds, are very often occasioned by intestinal irritation, instead of stone or stricture; and, "if the practitioner does not carefully distinguish these cases, he will become a party to the patient's fears and misconceptions, and expose himself, in the end, to the supposition of being either very ignorant of the matter, or guilty of a still worse fault. Were it not owing to the remarkable neglect of nervous sympathies which prevails; indeed I may say, without reservation, were it not to be attributed to the neglect of this part of pathology, I should have cases, not of mistakes, but of selfishness and dishonesty, to detail." It would have been more correct to have said, the almost fruitless attention to nervous sympathies, than the "neglect" of them; for the greater proportion of our modern books are full of pretended explanations of diseases on this principle. It is true that the subject has not been studied by all those who have used the term *sympathy;* which some of our inferior writers have at hand on all occasions, as readily as their histories of extraordinary cases and cures, though it is clear that they have no precise idea connected with it.

The ill consequences which result from the mistakes above designated, are then exemplified by the author in several cases. On coming to the rationale of these sympathies, he says, how they take place "it were quite needless to attempt explaining, unless my reader had accompanied me in the demonstrations of the visceral nerves:" but he adds, what may console pretty well such of us as have not enjoyed those advantages, that it is sufficient for practical purposes to know that such affections will result from irritation of the stomach, the ileum, and oftener the colon. The treatment of them must, of course, be agreeable with the indications for the remedy of the intestinal disease.

The fourth chapter treats of the "symptoms of the dilatable stricture, and of the spasmodic stricture," and displays in almost every page the great acumen of the author in clinical observation. The subjects of it, too, present several opportunities for an indulgence in a little quaint and very-pointed satire, to which he seems to be habitually inclined. Some respectable persons disapprove of this in treating on matters of science; but, whilst folly and knavery withstand grave admonitions, we ought to thank our stars that there are persons who will condescend to treat them in this way.

We can notice in a particular manner only some few points of this chapter; and, in the first place, the author's remarks on what is called the spasm in stricture. He first opposes the notion of John Hunter, that the occasional attacks of obstruction or retention of the flow of urine depend on spasm of the urethra itself: a notion which is founded on the supposition that the urethra has muscular fibres, which the author considers to be satisfactorily refuted. He attributes the occasional obstruction, not to spasm in the stricture itself, but to a spasmodic action of the muscles surrounding the urethra. This state of spasm, he argues, will often arise from irritation of the bladder, which is attended with a disordered relation between the action of the *detrusor urinæ* and the muscles of the urethra (enumerated on a former occasion), which are its antagonists. The natural relation, he says, is relaxation of the latter during contraction of the *detrusor;* but, in the case just mentioned, this relaxation is changed for sympathetic *contraction*. He introduces on this occasion a point of doctrine in physiology, which he has elsewhere stated, that the relaxation of muscles is not always a *passive* condition : he says, "If a finger or hand is extended or closed, it is not by the mere contraction of one muscle forcibly elongating its antagonist, nor does the relaxation of the one follow the contraction of the other; but, if the action be to bend the finger, the same influence which is exerted to excite the flexor to contraction, is also exerted to relax the extensor." He says, that the sphincter of the anus, and the orifice of the uterus, relax in this way, simultaneously with the contraction of the muscular coat of the bowels, the body of the uterus, respectively, and the abdominal muscles.

This doctrine cannot be easily rendered probable. Positive evidence of the supposed *laxative action* of the muscles is not obtained, nor likely to be obtained : the *necessity* of such an action is not rendered very plausible; and the inability of the expulsatory muscles in question to dilate or extend the sphincters in the natural condition of the parts, is not by any means demonstrated. This doctrine is, however, like many of Bordeu's notions especially, a little fascinating; for, by admitting it, we may conceive that we understand many things which are otherwise not intelligible : the very grounds, however, we must not forget, on which the belief in planetary influence on the affairs of men, supernatural omens, and other means of divination, have been established.

Stricture, the author says, is very apt, even when it is stationary, to disorder the natural sympathy of the muscles, and to induce contraction of the sphincter fibres; and these effects are more expressly liable to happen in stricture attended with inflammation. Inflammation of the urethra, too, without stricture, will lead to the same consequences. "Thus, out of a party of men drinking together, there will often be one who before the end of the evening cannot pass a drop of urine. On enquiry, it will be found that he has had slight disorder in the urethra, perhaps the remains of gonorrhœa. It is a mistake to suppose, in such a case, that the fulness of the vessels has closed the passage to the urine : the same cause which has inflamed his countenance adds to the inflammation and sensibility of the urethra; and the first drop of acrid urine is followed by contraction, and spasm and obstruction."

Various other analogous considerations are adduced by the author, which lead to the conclusions, "that the apparent changes in the stricture of the urethra are attributable to the disorder of the neighbouring muscles which surround the stricture, if it be seated within five inches of the bladder:" and, "as all strictures are accompanied by inflammation, which is severer at one time than another, they may all be attended with those symptoms which have been, by the greater number of surgeons, erroneously supposed to indicate a spasm in the stricture itself."

The fifth chapter is "on the cure of the dilatable stricture." As this stricture depends on inflammation of a spot of the membrane of the urethra, by which its natural elasticity is destroyed, and on which the inordinate action of the adjacent muscles depends, the first indication would seem to be to subdue this inflammation, and by means of low diet, blood-letting, rest, &c. These means are efficacious to a certain extent; but, the author says, "the patient may be long confined and greatly reduced, and, after all, derive but slight and temporary benefit from this severe treatment. In the use of the bougie, we have the means of applying pressure, and at the same time distending the inflamed membrane; by which, the low chronic inflammation is disturbed and remedied." The efficacy of distensive force alone, is shown by the fact that Bruninghausen used to cure stricture by directing the patient to use efforts to make water, whilst he stopped the passage of the urethra behind the glans. The irritation remaining after the use of antiphlogistic measures, may, however, the author considers, be removed more rapidly by using the caustic, not so as to produce a slough or abrasion of the part to which it is applied, but according to the indication of analogy in the use of caustic to the eye, to diminish the sensibility and irritability, and give a healthy surface even to an ulcer of the cornea. The argentum nitratum is that which the author employs. This mode of treatment is however advised only when "we find that, notwithstanding the use of the bougie, this troublesome spot of the urethra continues inflamed, and likely to be followed by worse consequences." The caustic can be applied in a much more exact manner, the author thinks, by means of the urethra probe, than by the common bougie. He has the ball of the probe made with a hole in it, in which a minute portion of the caustic is placed.

The formation of true, or permanent, stricture, its varieties and consequences, are next considered. This form of stricture is the consequence of that just described, and the almost certain consequence of it when of long continuance. The following is an abstract of the author's peculiar views of this affection.

The urethra is a dilatable elastic tube, but it has in itself no power of expansion. It is distended only by the force of the bladder and abdominal muscles, and by means of the urine flowing from the bladder. It does not naturally form an open canal, except when the urine is passing through it; at other times its parietes lie in contact with each other. Inflammation here, as in various other parts, changes the fine, pliant, elastic membrane, into a thick and firm structure, which gives more or less resistance to the passage of the urine or of an instrument: not because the urethra is contracted, for, in the words of the author, "the sides of the canal are always in contact, and cannot be brought closer by the formation of a stricture;" but because the elasticity, and consequently the dilatibility, of the part is destroyed. Inflammation is so constantly the forerunner of stricture, that "it may be held a point as well established by evidence, that the origin of all stricture in the urethra is in consequence of inflammation, as that adhesions of the pleura are produced by it." We are not acquainted with any valid objections to those opinions.

The most common cause of stricture, Mr. Bell thinks, is the inflammation attending gonorrhœa.

The author next describes the various appearances which stricture assumes in different cases and in different stages; and his opportunities for observation have presented him with some rare and interesting circumstances of this kind.

The effects of stricture are next discussed. The most common, and one of which the knowledge is of the highest importance in practice, is considerable dilatation of the urethra behind the stricture, from the inordinate impulse of the urine in this situation. This dilated part commonly becomes also inflamed, and sometimes ulcerated or ruptured; the consequences of which will be hereafter exposed. In all cases of confined narrow stricture, the author says, the prostate gland is found more or less affected: sometimes with inflammation and abscess; at others, such an enlargement of its ducts takes place, that the end of the largest bougie can be passed into them. Mr. Bell is inclined to believe that these cavities in the side of the urethra and prostate gland sometimes ulcerate, and produce the worst kind of fistulæ.

There is sometimes a very minute crop of soft warts growing from the membrane of the urethra, of which Mr. Bell has a specimen in his museum. This however is a rare occurrence, as Mr. Shaw has proved by his references to the best authors on this subject.

Thickening of the coats of the bladder, when the difficulty of passing urine has been long continued, always takes place; and there is often found, after death, signs of inflammation of this viscera, sometimes with spots of gangrenous ulceration.

The ureters are sometimes dilated "as large as a small intestine," and much inflamed; and analogous effects in the kidneys are occasionally produced.

The symptoms of confirmed stricture, the operation of sounding the urethra to ascertain the places and extent of the stricture, and the treatment by the bougie and caustic, are the subjects of the seventh chapter. The author's mode of treating these subjects,—that of describing first the nature of the disease, and its effects on the structure of the surrounding parts, and then stating the symptoms, topical and general, renders his account of these affections particularly perspicuous. Many of the topical symptoms are, indeed, so expressly indicated by the knowledge of the existing organic lesion, that it is hardly necessary to designate them in a particular manner; and the rationale of most of the rest must be readily understood. Hæmorrhoids and prolapsus, though a frequent consequence of stricture, are also, the author states, "of themselves capable of inducing those pains which are commonly symptomatic of stricture; and the disorder of the anus or rectum will sometimes cause an obstruction of urine;" of which Mr. Shaw gives a good example in a case related by Chopart.

Very good and precise directions are given by the author for sounding the urethra. This operation cannot be effected, in many cases, in so satisfactory a manner by the bougie as by the instrument of the author, which he calls the urethra probe. This instrument consists of a circular silver ball, with a wire, of less diameter, fixed in it. This is particularly useful when there are two or more strictures, as the ball moves perfectly free in the urethra, after it has passed the first, until it encounters the next; and consequently there is no danger of mistaking the resistance of that which it has passed through for another stricture, as is sometimes the case when the bougie is employed. The extent of a stricture can also, for the same reason, be much better ascertained with the same instrument. The precise seats of spots of morbid sensibility in the urethra, are also readily detected by the urethra probe; a thing which cannot often be accomplished by the bougie. The importance of bearing in mind the possibility of the state of dilatation of the glandular ducts in the posterior part of the urethra, is here insisted on; and the author refers to the specimens in the College museum and in his own, of false passages made by the entry of instruments into them.

The merits of the simple bougie as a remedy for permanent stricture, are then discussed. Mr. Bell remarks, that the too-sanguine favourers of the application of caustic in stricture have misconstrued the operation of the bougie in this disease, when they say it acts like a wedge merely, and dilates the passage; for, by the pressure of the bougie, an action is excited in the stricture, and the activity of the vessels adapts the form of the canal to the state of dilatation. The bougie, he adds, can never be laid aside in practice: in very many cases it is sufficient to remove stricture; and, even when caustic is employed, we also require occasionally to make use of the simple bougie. A series of excellent precepts for conducting the use of this instrument, then follow; and Mr. Bell concludes this section with the following passage, which coincides with the opinions and practice of the best modern surgeons, who are much experienced in the treatment of this disease.

"I have been long in the habit of using a silver instrument in the form of a bougie, [shaped like a catheter, we presume the author means,] further to dilate the stricture as soon as I have gained a certain degree upon the obstruction. The soft wax bougie I apprehend to be the best instrument while the stricture is narrow."

Having noticed the bad effects which may be brought on by the caustic, he remarks, with much force and quaintness,

"I believe this is equivalent to saying, that the caustic must never be used in confirmed stricture without a pressing necessity for it, and that it is to be used with the greatest precaution; and, having said this, I am at liberty to add, that it is a remedy which cannot be too much prized."

After treating on this subject with the minuteness requisite, the author discusses the important question of the propriety of "forcing the stricture" in certain cases. "This," he says, "is a most perilous subject to enter upon; full of difficulties; and especially when I consider it to be so much the disposition of the younger part of the profession to do things by force, rather than by patience and art, I am fearful of affording a precedent." It would be imprudent in us to pretend to give a satisfactory account of the author's discourse on this subject: every observation he makes is of essential importance, and any partial statement must give erroneous ideas of its meaning, unless connected with those which qualify the assertions it may contain. The author relates in detail one very interesting case, in which he resorted to this measure with the best success; and gives the abstract of another, of a similar kind. This section terminates with the following remarks.

"All that I find myself confident in concluding is, that when there is a stricture of the nature of a little filament, (and such is the stricture most commonly found when there has been no previous violence committed,) we may force it, if there be serious and pressing reasons for immediate operation. But, on the contrary, where the stricture, by frequent returns of inflammation and much labour with the bougie and caustic, has become a dense body, the attempt to force the passage with the catheter will not succeed, and may be fatal."

It will not succeed, because the urethra will give way rather than the stricture. Mr. Shaw accompanies this section with some very good remarks and apposite cases.

A question not inferior in importance to that just alluded to then arises, that of the time and mode of puncturing the bladder, in cases of partial or total obstruction from stricture. Justice cannot be done to the author's considerations on this subject in an abstract. Mr. Bell zealously favours the practice of opening into the urethra behind the stricture, when an operation of this kind for the relief of the bladder is necessary. He says,

"This operation, when performed for a stricture of the urethra, (not curable by other means,) and when there is no complication nor destruction of parts by the extravasation of urine, is perfectly successful. I believe this manner of relieving the bladder would be more followed, if surgeons were aware, by as many proofs as I have before me, that the membranous part of the urethra is always dilated in stricture."

Another difficult case in this department of practice, is that in which the patient is wasting rapidly from exhaustion, occasioned by an almost incessant call to make water as fast as it comes into the bladder. Here the bladder never rises high enough to be punctured above the pubes; and it is difficult, if not impossible, to puncture it by the rectum.

"In such a case," says the author, "I hold myself authorized to cut directly upon the stricture, and to open a passage into the urethra behind it. By relieving the bladder from the necessity of violent and frequent action, the cause of the thickened coats is removed; they relax, and permit the accumulation of urine. Whether we are to attempt the further destruction of the stricture, and the restoration of the natural course of urine, just at this time, will depend on the strength and resolution of the patient."

In cases of retention of urine, when the bladder has risen above the pubes, and when it is probable that the natural passage may be restored in a short time, as a few days, puncture of the bladder itself should be effected. The place and manner of operating must be further determined by the nature of the obstruction; but there are very few occasions on which the author, with the generality of surgeons, does not think the operation by the rectum preferable to that above the pubes. Mr. Bell has hitherto performed this operation in the ordinary way; but he says,

"I have determined, if it shall be necessary on any future occasion to perform the operation in a young man, to change the mode of operating. My reasons are these: on dissecting the parts, after death, I have found the canula introduced betwixt the vasa deferentia so critically as to touch them both! On dissecting the parts in the natural state, we must observe, that it can only be by a happy chance that the prostate, the vesiculæ seminales, and vasa deferentia, escape in this operation; and, in fact, I possess a preparation where the bladder was four times punctured, and each time some of these parts were wounded. A very little variation in the manner of operating will avoid these dangers. When the finger is introduced in ano, and a proper distinction made betwixt the membranous part of the urethra, the prostate, and the body of the bladder, let the trochar be introduced, not exactly in the centre and behind the prostate, but to one side of the prostate. If the bladder be struck behind, and a little to one side of the prostate gland, the vasa deferentia would be then distant from the wound. The vesiculæ seminales would also be avoided; and we have this further motive, that, if they were touched, the wound of them would be attended with no serious effect, while the division of the vas deferens would be equal to castration."

Mr. Bell adduces one argument in favour of puncturing the bladder above the pubes, which should be borne in mind in all cases,—that of the opportunity it affords of piercing the callous portion of the urethra: by introducing a catheter through the wound into the bladder, then into the urethra, and by pressing it up to the back of the stricture, it would facilitate any operation we may choose to perform on the fore-part of the urethra. He adds,

"Although I object to the cutting or piercing of a stricture, in common circumstances, with an instrument passed into the urethra, yet, were I to be so happy as to have an instrument at the back of the stricture, I should have no hesitation in passing a catheter, with a stilette, into the urethra, for the purpose of piercing the stricture. I would proceed thus: having carried down the catheter to the stricture, I would push out the stilette, and lodge it in the extremity of the catheter which had been passed from the bladder; and, thus covered and protected, and directed by the upper instrument, I would pass it into the bladder."

The subject next treated of, is "the bursting of the urethra."

"This," the author says, "is very properly considered as belonging to the higher department of surgery; that is to say, it treats of cases which require the most perfect acquaintance with the principles of the art, and a dexterous and practised hand; one not partial to operations, yet not hesitating to do what appears bold, when the occasion calls for it. An undecided conduct, vacillation, and half-measures, have lost patients in the case of ruptured urethra; and I am the better entitled to call my reader's attention to this necessary decision of practice in the present instance, since, on former subjects, I have, I hope, taught him to rely on gentle measures and perseverance. But this boldness is a seducing word, and may very well be allied with ignorance. To make this boldness decision, he must be perfectly acquainted with the principle which is to guide the surgeon on this very trying occasion."

When a patient has had a stricture attended with much irritation in the perineum and neck of the bladder; when he has to strain and force to pass a few drops of urine; when the urine feels scalding hot; and when the patient, on closing his legs, has a sensation of a tumor between his thighs, though there be no tumor there; then, the author says, he is in danger of an extravasation of urine from rupture of the urethra. These symptoms will come on suddenly in some cases after the rude introduction of a bougie, which is followed also by rigor, fever, and strangury. At length, after the symptoms just described, the patient, on straining hard to make water, feels that it is flowing; but it does not appear outwardly, although the bladder is relaxed; it flows into the scrotum; and, after a little time, the scrotum becomes distended to a considerable size. The patient is seized with shivering low fever; and, if the swelling be not immediately relieved by free incisions, the integuments of the penis become distended; the urine then extends to the integuments of the pubes, of the abdomen, and even of the loins. A dark-coloured inflammation then affects the skin of those parts; the skin and subjacent cellular membrane slough; the whole scrotum separates, and leaves the testicles exposed. If the patient be not well supported by stimulants at this time, he sinks, and dies, with all the symptoms of the influence of mortification on the constitution.

The rupture of the urethra takes place in the portion of this membrane situate behind the stricture; which has been already stated to become distended to a great size, and sometimes ulcerated. The urine is directed forward to the scrotum by the fascia of the perineum.

The author relates ten cases of rupture of the urethra, of the kind just described: in five instances the patients recovered, chiefly by means of free incision into the parts distended with urine; the use of bark, wine, or brandy, and good nourishment; anodyne fomentations, camphorated spirits, &c. to the gangrenous surface; and the introduction and retention of a catheter in the bladder as soon as it could be effected. The others died; and some of them, in all probability, from the neglect of those means, especially of the free incisions. To this concise abstract we may add, with much advantage, the following general remarks of the author, in his own words.

"1°. It appears that punctures of the scrotum are insufficient even to empty the cellular texture of the extravasated urine, and quite unfit for preventing the urine taking the same course a second time. If the lancet be used, the shoulder must be moved, while the point is kept at rest, so as to make a large opening in the skin.

"2°. For the most part, the urine bursts into the perineum, and is carried by the fascia of the perineum forward into the looser scrotum. In this case, the opening into the scrotum must be at the back part, and the point of the instrument directed backwards, so as to cut freely through the fascia, and give issue to the urine as it escapes from the perineum.

"3°. But it will be seen here, that the extravasation takes place sometimes more anteriorly, and the *œdema* of the preputium is the first sign of the approaching danger. In all cases, therefore, it is proper to sound the urethra with a bougie, (and this should be done in the gentlest manner,) to ascertain the place of stricture, that the puncture may be directed with reference to the spot from whence the urine issues from the urethra, and which is always behind the stricture.

"4°. The urine has a deadening effect on the cellular membrane, when it is permitted to fill the integuments. When in a smaller quantity, and with diminished force, it produces a blush of erysipelas, which subsides, and rises again in the form of more phlegmonous inflammation."

The last-mentioned affection requires a different treatment from the gangrenous disorder: it should be treated with cold and sedative applications. Here the distinction of effects is of great importance. It sometimes happens also, the author adds, that, "after we have made a free passage for the urine, that the integuments of the penis are again puffed up, as if the urine had again been forced into the cellular texture: but this is an effusion of another kind; it is serous, and is a consequence of inflammation." The same appearance will also occur without any extravasation of urine. Cold applications are best when it is arising; but tepid fomentations should be used when it is in a late and aggravated state.

"If the nature of the swelling should be mistaken, (and, being often complicated with stricture, it is apt to be mistaken,) and if a catheter be introduced into the bladder, under the idea of carrying off the urine, then the swelling will increase, and terminate in suppuration of the integuments of the penis."

Under the head *Urinary Abscess,* the author considers "all those abscesses which are occasioned either by irritation within the urethra causing abscess external to it, or by the escape of urine through ulceration of the canal; for, it will be remembered that the urine does not always escape at once abroad into the cellular membrane, as it does in the cases of *burst urethra.* It will now appear that sometimes it makes its way by a little at a time, and by the irritation of its presence produces abscess." Several cases of this kind are related in detail; and then the author passes on to the consideration of the "operation for fistula in perineo;" an operation sometimes requisite in the cases last alluded to; that is to say, on those occasions where the stricture cannot be removed or much relieved; or where, from other causes, the orifice of the fistula has been for a long time in a callous state.

The treatment of cases of retention of stones in the urethra, is next discussed; chiefly in regard to a description of the various means which may be resorted to in different cases to obtain their expulsion.

Several cases of "rupture of the urethra by violence," are next related; and then the author treats "of the diseases of the rectum," or rather some of the affections of this part; for he professes to describe none but those with which he is familiar, and no operation which he has not repeatedly performed; and his motive for writing these observations is, that he believes he has noticed "some circumstances in the performance of the operations on this part, which, if attended to, will render them more safe, and not less certain in their beneficial effects." These are preluded by some remarks on the action of the muscles connected with this part, which the author considers qualified to elucidate the nature of some of its diseases; the chief of which is, that the levator and the sphincter of the anus are not antagonist muscles, and that they both tend to prevent prolapsus of the anus on the expulsion of the feces: the one contracting around the extremity of the gut, and the other drawing it up and sustaining it. The author, reasoning on the foundation of his notion that the relaxation of sphincter muscles is an *active function* in the muscles themselves, says, that, "without a certain state of the rectum itself, these muscles will not relax. By the exertion of the will over the muscles of the abdomen and diaphragm, the viscera are powerfully pressed down; but the feces are not expelled: the levator and sphincter retain their activity, just as they do during the common state of exertion of the muscles of the belly, as in crying or speaking, when they remain forcibly contracted round the orifice of the rectum." Instead of this being an illustration of the truth of the author's notion of the active relaxation of the sphincters, we think it at best but a very weak argument for it: still there is so much ambiguity in it, that it cannot be positively refuted. We all must know that a man *can* expel at will feces from the rectum at any time when they are present in it, when no natural inclination to expel them had been present, unless they are preternaturally hard; but here, it may be said, the "certain state of the rectum" does exist, and here the dispute must then end in the author's favour; and so must every dispute terminate, if gratuitous explanations conformable with conjectural assumptions are suffered to take the place of proofs of the premises. The author adds, that "nothing tends so effectually to disorder the connexion of the sphincter and levator muscles, in the act of expelling the feces, as the absorption of the fluids of the rectum, and the lodgment of the hard feces in the lower part of the intestine. Then it is that the patient may strain ineffectually; for the rectum does not contract, the sphincter does not relax, the levator resists, and the only effect produced is the distension of the veins of the verge of the anus, and the forcing of the upper part of the rectum to descend into the lower and more capacious portion." This explanation is consonant with the principles just noticed: other persons may prefer another, requiring less conjectural reasoning, namely, that the force which will expel soft feces from the intestinal tube may not be sufficient to expel hard feces, considering that this tube lies in folds, that the expulsive force is not directly from behind, and that the diameter of the cavity of the tube is greater than that of its orifice, the anus, in a state of moderate distension.

As certain states of the rectum will not, according to the author's doctrine, be attended with sympathetic relaxation of the levator and sphincter of the anus, so certain other states of it will be attended with inordinate sympathetic relaxation of those muscles; thus, he says, "if any peculiar irritation takes place within the rectum, by which the relaxation of these muscles is excited, they lose their guardian office, and let the inner membrane of the rectum descend, and permit the veins of the anus to be distended."

The author cannot, we presume, have intended to advance the above doctrine as any thing else than *hypothesis*; though he has stated it with the imposing character of *theory*, or, more properly speaking, he has produced it as exempt from any doubting, or want of assurance, as any of the theoretical reasoning in the volume. Hypothesis may be of beneficial utility in medicine, as well as in other sciences, by indicating the routes for further acquisition of knowledge; but it is wrong to attempt to make it assume a false character, and confound it with theory; for it is such conduct as this which has caused so much unjust odium to be accumulated on medical theory, as exists in the present day.

The diseases noticed in this section by Mr. Bell are, warts within the verge of the anus;—small fistulæ on its margin, which the author is inclined to believe are the result of inflammation and irritation of the glands which lie under the fine skin of the anus;—stricture of the orifice of the rectum, without and with hemorrhoids. The use of the bougie in diseases of the rectum and anus, and stricture of the rectum, are then discussed. The schirro-contracted rectum is afterwards treated on. On these various subjects the author advances but little of importance that is peculiar to himself. Relaxation of the inner membrane of the rectum; prolapsus of the anus; soft tumors in the rectum; and hemorrhoids; are then considered. The first affection produces invagination of a superior in an inferior portion of the intestines, and is compared with the *intro-susception* of the higher parts of the intestines, and is attributed to excitement and irritation of the gut. Prolapsus of the anus is said to arise from the same cause, and not from relaxation. It is frequent with children, the author says, "from the nestling of ascarides in the gut." There is a more chronic form of this affection in adults, consequent on long-continued or habitual costiveness, which arises from a previous distension of the gut, from which the folds of the inner membrane lie very loose when the distensive agent (accumulated feces) is removed, and are protruded by the efforts to go to stool; the protrusion being favoured by "a relaxation of the sphincter," with which this affection is accompanied. "When this consequence of torpor of the bowels has continued for a long time, the protruded fold of the rectum is elongated, and assumes something of the character of a tumor, when inflamed and turgid, but of a shrivelled membrane, when not swollen and inflamed." In the latter state, we may with propriety, the author says, "cut off these pendulous flaps of skin. I have done this operation by taking hold of one of the flaps with my finger and thumb, and passing the needle through it near its base, then entering the ligature so as to make two: these being tied, one on each side of the flap, the projecting part is snipped off with the curved scissars. The ligature and remaining part of the flap are left to be withdrawn within the gut. A starch glyster, with laudanum, should be given in the evening, and the patient must keep to his bed or sofa for several days."

The author next treats of hemorrhoids, and the tumors connected with them; and, on speaking of the operation for them, he remarks that it "may be done on the relaxed and shrivelled pile, in its chronic state; but it is not to be thought of, when there is a tense and painful tumor occupying the margin of the anus." The tumor to which the author here alludes, is the result of inflammation of the mucous membrane of the gut covering the pile, and perhaps of extravasated blood beneath it, around the distended vein also. The mode of operating practised by the author, is similar to that just described as proper for the pendulous tumor formed by the inner membrane of the rectum.

"Fistula in ano," is the last subject for illustration in this volume. The author first distinguishes abscesses in this situation into those independant of any disease of the rectum; and those connected with irritation, or some other affection of the intestine: each of which species is subdivided into several varieties. He dwells a little on the interesting fact, that a state of irritation or inflammation of the rectum, as well as of the membrane lining the fauces, the lacrymal duct, the trachea, and the prostate gland, will lead to abscess in the surrounding cellular membrane, though the rectum itself may not be in a state of suppuration; and this is a fact of very great importance in respect to practice; for it must be evident that all attempts to cure these abscesses must generally fail, whilst the original cause, as piles, warts, stricture of the rectum, &c. continue to exist. Although the abscess is at first distinct from the gut, yet the latter part commonly at length ulcerates, if effectual remedies are not employed; and thus the complete fistula is formed. This communication between the cavity of the intestine and the abscess may take place either before or after the abscess has burst externally; the former occurrence constitutes what is sometimes called blind fistula. The volume terminates with a description of the several operations which may be practised for the cure of those affections. Mr. Bell does not say any thing about the origin of fistula in the pouches of hemorrhoids, as described by Dr. Ribes, of the Hospital *Gros Caillou* at Paris, of which an account was given in a late Number of this Journal. Mr. Shaw adduces the observations of M. Ribes on the situation of the internal orifice in the generality of cases, but makes no allusion whatever to his statement of the origin of the fistula. Mr. Shaw's intention seems, however, to have been to bring forward only such observations and opinions of other surgeons as are either conformable with those of Mr. Bell, or such as he thinks Mr. Bell has shown to be erroneous. When a writer has fulfilled his intention, there is no reason for complaint; but we think Mr. Shaw might have extended his object with some advantage to a comprehension of such observations of foreign authors as are apparently true and useful, and have escaped the notice of Mr. Bell.

An appendix to the work contains a description of the preparations in Mr. Bell's museum, of parts connected with the diseases which are the subjects of the foregoing disquisitions, and which are occasionally referred to in proof of the author's observations and opinions.

*A Treatise on Derangements of the Liver, Internal Organs, and Nervous System.* By JAMES JOHNSON, M.D. Author of the "Influence of Tropical Climates on European Constitutions." Third Edition. 8vo. pp. 223. Underwood, 1820.

THIS work, which is called by the author a Treatise, might with more propriety have been named a Dissertation: it is obviously imperfect for a *treatise;* being little more than a medical rant, in which the practitioner who seeks to have his information improved on any one subject on which it treats, will look in vain for instruction or assistance. It is a laboured rhodomontade, which may be profitable to the author if it gains him reputation, no matter how deserved; profitable to the bookseller, if it sells; and will indubitably become profitable to the venders of candles, tea, and butter; tobacco-smokers, physic-takers, and all others who may be interested in purchasing waste-paper by weight.

Ungrateful as is the task, the duty has nevertheless devolved upon our wretched selves, of perusing carefully these still more wretched pages, and of making out of them a dish for the *entertainment* of our readers, since, from the badness of the article, their *instruction* is entirely precluded.

The volume commences with general observations on sympathies; then follow five sections: 1st, on diseases of the respiratory organs; 2d, gastric complaints; 3d, cutaneo-intestinal sympathy; 4th, on derangements of the hepatic system; 5th, diseases of the heart: to which are added, observations on the nervous system, (a sort of interlude,) and an essay on hygiene, or conservation of health, including nine sections: 1st, air; 2d, food; 3d, drink; 4th, exercise; 5th, clothing; 6th, ablutions, bathing, &c.; 7th, the passions; (diseases of literary persons in a parenthesis, not enumerated in the sections;) 8th, sleep; 9th, medicine.

The first part of the work consists of introductory observations on the atmosphere, lungs, vicissitudes, powers of constitution, skin, &c. The author then favours us with an account of certain sympathies between the skin and other parts. He enumerates "a cutaneo-pulmonary sympathy, a cutaneo-gastric sympathy, a cutaneo-hepatic sympathy, and a cutaneo-renal sympathy. This enumeration of the sympathies is certainly very ingenious, and we are willing to allow the author all the merit he claims for such discoveries: we would however suggest to him, with much diffidence, that we have known exposure to cold followed by inflammation of the brain, or of the eye; also by swelling of the testicle, also by enlargement of the parotid gland, also by pain in the loins, and also by inflammation of the toe. In order, therefore, to complete the account of sympathies which he has so ingeniously discovered and sketched, we would submit, with great deference, the following additions to his list; namely, a cutaneo-cerebral sympathy, a cutaneo-ocular sympathy, a cutaneo-testicular sympathy, a cutaneo-parotidean sympathy, a cutaneo-lumbar sympathy, and a cutaneo-digito-pedal sympathy. Perhaps future observation may develop additional sympathies of this class. We should not omit to mention that the author's treatise professes to be a *practical one*, as will be sufficiently obvious from the general exhibition of its contents, to which we now proceed.

The sympathy between the skin and the lungs, is thus illustrated and explained. "On immerging the body in water many degrees below the temperature of the skin, the vessels on the surface *are struck torpid*, and the blood is determined to the interior. At this moment *a sympathetic torpor* takes place in the capillary vessels of the lungs, so that the blood is with difficulty transmitted through them, *occasioning that panting* for breath which we observe in all, but more particularly in delicate people, at the instant of immersion." We have the author's word for all this, and no doubt it is very true. The manner in which cold produces consumption, is said to be by "exciting scrofulous tubercles there (existing in the lungs), into a state of inflammation."

The author takes occasion to infer a cutaneo-gastric sympathy, because it has happened that a person has had a pain in the stomach on putting the feet into cold water: hence, from this sympathy, heart-burn, flatulence, &c.

Heat, the author asserts, produces a synchronous increase of perspiration and of biliary secretion. At the same time, it is confessed that the general effect of an increased determination to the surface, is to diminish the determination to, and the secretions of, the internal glandular system. The author omits to give any satisfactory reason for his opinions, trusting, we presume, to the weight of his authority, which is no doubt quite sufficient. Then follows a *recapitulation* of the previous absurdities, the object of which is to impress them on the reader's memory.

One of the first symptoms of asthma, is said to be "a sudden inclination to stool;" which, as the author observes, is certainly *very remarkable.* The following, to do the author justice, is a full and beautiful description of the symptoms of asthma. "It is remarkable that the first *warning* symptom is often a sudden *inclination to stool*, or intestinal irritation, which as quickly *shifts its seat to the stomach*, and then *flies to its favourite station, the chest.* Now come on convulsive dry cough; difficulty of inspiration; heaving of the chest and shoulders; wheezing; *pallor or livor* of the countenance; inability to lie down; coldness of the extremities; gasping for *fresh* air; great perturbation in the pulse, which is *weak* or *throbbing*, *intermitting* or *redoubling*. Head-ache next succeeds in most people; feverishness; belching of wind, and discharge of the same downwards, especially when the paroxysms *are taking the turn.*" The author has forgot to exclaim how *remarkable* it is for *an inclination to stool* to take its seat *in the stomach*, and then *fly to the chest.* Then follows an almost equally pathetic description of consumption, in which are the following phenomena. "The hectic flush on the cheeks, the vermilion lips, the burning heat in the palms of the hands and soles of the feet, with evening fever, are periodically changed for cold colliquative sweats, hollow, pale, languid countenance, sharpening features, augmented expectoration, and progressive emaciation." The account goes on to speak of "heart-rending symptoms, agonized friends, and the never-dying hopes which perpetually spring in the hectic breast." Poetical as this description may seem, we have nevertheless pleasure in giving our testimony to its general truth; for we have ourselves before now seen the very things which the ingenious author has just described.

Cold is said to improve the appetite and health of the stomach, (p. 26;) and a little before, the author devotes a section, to the end of showing that cold produces the worst effects on the stomach, by means of the cutaneo-gastric sympathy. These different effects, however, the author imputes to different degrees of torpor of the skin.

It is asserted (p. 28), that disorders of the internal viscera, as the liver, stomach, &c. *are not inflammatory*, *unless produced by climate;* which must be a great consolation and encouragement to dram-drinkers.

*Obstinate costiveness* is given as the characteristic symptom of inflammation of the *external coat* of the bowels. We must therefore have been frequently mistaken in finding peritonitis accompanied with a diarrhœa.

The account of dysentery is freer from objections than some others; at the same time there is nothing to praise in it: it is such as an indifferently-informed apprentice might dictate; and here, as well as in the succeeding topic on cholera morbus, a wretched passion for hypothesis prevails, of which, as a sample, let the reader take the following explanation of cholera. "If, therefore, we consider that the functions of the skin and the liver are inordinately increased during the heat, and, of course, rendered more liable to a check on the application of cold, we shall easily conceive how a sudden atmospherical vicissitude, at this period, by *inducing a torpor* of the perspiratory vessels on the surface, and of *the secretory vessels of the liver* sympathetically, will *unhinge* the *balance* of the circulation and excitability; and that, while a great plethora is induced throughout the *portal and mesenteric circles*, the torpor in the liver and surface is succeeded by an increased irritability of the stomach and intestines; and that this increase of excitability in one organ, or set of organs, while the excitability of others is decreased, is the mean by which nature effects the restoration of the balance." We hope our readers will be as fortunate as the author, and easily conceive all this.

We have not given any account of the treatment the author advises in the foregoing diseases, because we apprehend that such an exposure would lessen the zeal of the reader's meditation on the ingenious doctrines which have just been put forth: for we should have had to tell the story of what we saw on being called to a patient having dysentery, who had been treated according to the instruction given by this same author only two years ago, in the second edition of this work; which was by "the exhibition of submuriate of quicksilver, in scruple doses, twice or three times a-day, without any other medicine; a remedy discovered by him, that is Dr. Johnson, in the East Indies, and which he has employed "in dysenteric cases occurring in this country, with much felicity of result:" (2d edit. p. 47.) and it would have been very unkind in us to have hinted at the reasons which may have led him to omit mentioning these *felicitous* results in the present edition, and to substitute for the remedy he discovered, that of "*a grain of calomel*, half a grain of opium, and a couple of grains of the pulvis antimonialis, every six hours." (3d edit. p. 41.)

The subject of hepatic diseases, is prefaced by a physiological disquisition on the function of the liver and the use of the bile. The author here, as on all other occasions when he does not utter absurdities, is a mere compiler; and we cannot compliment him on doing this easy duty with much judgment, as he generally selects for his assertions just those points which stand most in need of proofs, which he seems to think quite unnecessary. He takes every thing for granted which suits his convenience, and if he wanted any thing else, he would not hesitate to assume it. Among other examples of a mixture of gratuitous assumption, conjecture, and assertion, is the following. "These fluids, (pancreatic and biliary,) when poured on the chymous mass, penetrate, dilute, and *animalize* it. They, in all probability, are the principal agents in separating the chyle from the excrementitious matter; that is, in *precipitating* from it whatever is not nutritious. In this process, the bile itself *seems to divide* into two portions. Its oily, bitter, and colouring matter, combines with the excrement, and possibly imparts to it those stimulating properties, by means of which it excites the peristaltic action of the alimentary tube; while the other constituents combine with the chyle, and re-enter the circulation." It is admirable the facility with which the author disposes of the two portions of the bile, the existence or separation of which he *only fancies:* it would be vain to task his ingenuity or his wisdom for the evidence which should confirm such a point of physiology: we might as reasonably expect to draw blood from a flint.

A case of hepatitis is given, said to have been caused by a blow on the side. It occurred six months after the accident, got well, and occurred again in six months more. Several months afterwards the patient had another attack, owing, as it is said incidentally, "to intemperance and exposure to cold." Now this is given as a case of inflammation and abscess of the liver from an accident, which had occurred many months previously; the influence of *intemperance* being overlooked as a cause in the first instance, because then it would not have been the strange case which it is denominated: but the intemperance is brought in afterwards as a very sufficient reason for the patient's having not only hepatitis, but an abscess of the liver terminating in death. This is one among many hundred instances of the arrogant assumption of the author. He perceives no difficulties, and drives on helter-skelter, with a random prosing, in which he explains every thing, and always in his own way, and seldom or never correctly.

We are informed, that "there is no *symptom* during *life* by which we can positively ascertain that tubercles are forming in the liver." Will the author, in his next edition, inform us by what symptoms this is ascertained after death? Then follows a selection, with nonsensical remarks from Drs. Baillie, Farre, &c. on the subject of tubercles in the liver, by which it appears that the liver is liable to tubercles which are not all alike: but then we can neither know that they exist before the patient dies, nor cure them if we could know of their existence; for which reasons it is unnecessary to say much more about them. The author nevertheless, with his usual boldness, does give a plan of treating tubercles of the liver, notwithstanding he had just confessed that their existence could only be ascertained after death. These are delightful impertinencies. It is thought probable "that *stagnant blood oozing* from the liver *finds its way* into the *intestines*, producing melœna, &c." When a person feels creeping chills, is pale, and has cold feet, let him conclude that he has congestion of the liver; for by these symptoms it is said to be characterized, with, it is added, "in short, all those common phenomena of venous congestion;" which common phenomena our author, very consistently, omits to mention: possibly for the reason that he does not know precisely what they are.

The author next exercises his fancy in enumerating eight consequences of deficient secretion of bile; and concludes his matter-of-fact statement by observing "that, partly from sympathy, and partly from deficiency of bile, a morbid excess of irritability accumulates in the nervous system, which *inequilibrium* of excitement explains, in a great measure, those *mental symptoms* accompanying a disordered state of the biliary and digestive organs:" and we may add, explains them at once beautifully, pathetically, and satisfactorily. The physical virtues of Cheltenham and Bath waters (an odd association), are said "to depend on *their warmth* and *purgative* qualities"! The author next informs us, "that whenever hepatic derangement amounts to any thing like *organic lesion*, we feel *in general some local uneasiness*, either in the region of the liver," or somewhere else. From the use which the author makes perpetually of the word *lesion*, it is evident that, as a medical term, he does not know the meaning of it.

Several pages are next devoted to an account and recommendation of the nitro-muriatic acid bath. Of this nostrum we have had no experience, and that which is reported to us is so very defective, as, in our estimation, to carry no weight. We would be understood neither to praise nor censure a proposal, the merits of which have not been investigated. At the same time, we have very little confidence in things of this description, which have been proposed and panegyrized in all ages; have served to get a little reputation for their inventors, and then have sunk into oblivion. Our general experience of nick-knacks of this description, is therefore unfavourable to the introduction of new ones; and then, the following contradiction in the lucubrations before us does not increase our confidence. At page 113, line 7, Dr. Johnson informs us, "that the nitro-muriatic bath *purges*, and gives rise to the expulsion of dark-coloured feces, &c." and at line 17, same page, he tells us "*it is necessary to keep the bowels open during the use of the bath.*"

The section on diseases of the heart, is prefaced by some observations on the powers and mode of the circulation. The much-disputed question of the share of the heart in this business, is touched upon. The author here seems to find himself in an involved subject, on which individuals of talent have entertained different opinions. He himself, that is to say the author, Dr. Johnson, does not seem to have any opinions, and he goes over the ground gingerly, "like a cat upon hot bricks." We nevertheless congratulate him upon the success with which he has steered through all conflicting opinions; the adroitness with which he compliments opposing writers; and the flattering coincidence which he evinces with the sentiments of all. He is quite clear upon one point, which is, that the circulation of the blood through the veins is not performed by the surrounding muscles. We thank him for this information. The author is strongly disposed to think "that the capillaries are possessed of *an erectile power*," to which he is inclined to impute a considerable share of the circulation of the blood. As this theory seems ingenious, we wish he had enlarged a little upon it.

The symptoms of diseases of the heart are then enumerated under seven divisions, which are ycleped primo, secundo, tertio, quarto, &c. but which contain nothing remarkable, except the erudition displayed by their enumeration, together with some picturesque descriptions. Dr. Johnson thinks *tailors* more subject to diseases of the heart than any other men: at least, this is the result of his experience; the amount of which has not been stated. This seems an unvarnished fact, an original observation, which shines brilliantly amidst the darkness which an indifferent taste in plagiarism has contrived to throw over the subject. Some cases of carditis are given; chiefly copied, and the authorities acknowledged. There is one, however, the authority for which is not mentioned, and which the author would let pass for his own, and perhaps it might be his own; though, as he is not sparing of credit to himself on all possible occasions, it may be doubted under whose authority the second case is reported.

The symptoms of carditis, like those of other internal diseases, are very equivocal; there are circumstances in common to this and other affections which render a satisfactory diagnosis difficult, sometimes impossible, to those who are well qualified to make such distinction, both by their opportunities and discernment. We therefore did not expect from our author much instruction on this subject, and we are therefore not disappointed in not finding it. After filling many pages on the diseases of the heart with a bad compilation from the popular writers on this subject, the author observes, that there is considerable difficulty in distinguishing organic diseases from mere functional disturbance of the heart. This difficulty has been pretty generally acknowledged. The least exceptionable criterion is perhaps furnished by the permanence or occasional cessation of the symptoms: diseases of structure act constantly, and therefore produce symptoms which are equally regular, liable indeed to varieties of degree; while, in diseases of action only, there are often complete suspensions of their phenomena. This distinction, which is a very common one, appears to have been overlooked by our ingenious author: he, however, says something about "the purring of a cat," (p. 154,) which may serve to compensate the omission. He also informs us, that he knows at this time four men with diseases of the heart: we regret that he has omitted to mention how many of them are tailors.

The author next expresses his intention of illustrating his observations by two cases, "not (he says) copied from imagination, but from nature;" and immediately commences his description of two imaginary cases. Thus, "we *will suppose* that ossification of the valves, or dilatation of the pulmonary cavities of the heart, takes place, accompanied by," &c. He says that "a hand accustomed to thoracic percussion can tell how far the liver rises under the ribs; while the abdmoninal compression in various postures ascertains *its dip*" (in italics, *its dip!* certainly a very pretty phrase; a quaint, emphatic, pretty, phrase: the author is therefore right to put it in italics,) "in the belly." This is no doubt very clear to our ingenious author; for ourselves, plain men as we are, we are so obtuse as to regard it as little better than nonsense; a commodity of which the author is not sparing. The section on diseases of the heart concludes with a recommendation to examine *the urethra*, from which unsuspected quarter the author has found diseases (we presume from the context) of the heart, liver, and lungs, to proceed.

We are next presented with an episode entitled "observations on the nervous system," which is introduced by an attempt at a classification of nervous diseases, in which those of motion without sensation, are omitted. This is an unlucky attempt at originality, in the failure of which we sincerely sympathize with our patient author; for such attempts must cost him considerable efforts, and, as they occur but rarely, 'tis a great pity that they should always miscarry. The episode proceeds, imperfectly following the above-named imperfect classification; and in the midst of it is a quotation from Broussais, occupying some pages, followed by quotations from Whytt, and by appropriations of bits and shreds of doctrine miserably made into a sort of patch-work, the original owners of which are not confessed. And these are observations upon the nervous system! a mass of the most consummate jargon; a collection of the worst materials, put together without taste, method, or judgment; a string of unmeaning sentences, fitted only for the delusion of fools. Not a fact, nor an observation, or even an intelligible theory, for the mind to rest upon, except those mutilated shreds of statements and opinions, which are so clumsily quoted, and so imperfectly put together; some of which, certainly, deserved better than to be so profaned. We would preserve our good-humour, and laugh, if we could, throughout, at this specimen of book-making; but our patience is almost exhausted; and, if we should perchance sometimes appear a little splenetic, a reader of any conscience who should peruse this volume would hold us excused.

The next and (thank God!) the concluding division, is entitled "Hygiene, or Conservation of Health," &c. It begins with a lamentation, the substance of which is, that artificial man won't live like natural man. It appears from this section, as well as other parts of the work, that night air is bad, hot air is bad, cold air is bad; clothes too warm are bad, clothes too cold are bad. This division of the work further asserts, that there are five circumstances to be attended to on going out into the night air: it recommends a muffler and other things; and we are surprised that our learned author did not make a special paragraph in favour of clogs: nothing is said either about spencers, or woollen night-caps; but it is affirmed, by way of substitute, (in a quotation,) that men's minds, like barometers, are governed by the weather; "when the weather is rough, turbulent, cloudy, stormy, &c. men are dejected, angry, waspish, dull," &c. Then follows a quotation from Virgil, tending to show that he entertained similar sentiments; for which honourable notice, Virgil's ghost, and all the other authors who are mentioned (chiefly modern ones), and their ghosts, ought to feel greatly flattered.

On the subject of eating, drinking, &c. the author recommends a pretty hearty tea or coffee; and favours us with the information, that the stomach requires *repose* as well as the voluntary muscles. The author is very poetical on the article of drink: he draws pictures with much force and pathos, and deals out profound observations with so prodigal a hand, that one would almost think they were of no value, which would be a great mistake. Malt liquors, he asserts, "fill the blood-vessels more than any other species of drink: *they paralyze the absorbent system*, and render *torpid* many salutary *secretions*." The reader cannot fail to admire the charming imagination of the author. Tea is either good or bad, the author does not tell us which, precisely; but he indulges us with a rhapsody, which will do as well.

It appears, from the chapter on Exercise, that too much exercise is bad, and also that too little exercise is bad. The author recommends moderate exercise as being most conducive to health; a piece of advice for which we ought to be eternally grateful: to say nothing about our admiration of the genius which made so brilliant a discovery.

Clothing should be of a reasonable kind; and the author does not think it wise to adhere too closely to *the fashion*, which he facetiously calls "a tyrant;" and a pretty metaphor it is.

Previously to the recommendation of cold-bathing, the author advises the chest to be examined by percussion. He thinks that cold-bathing is good under proper circumstances, and that it is bad under improper circumstances; and he entertains the same opinion of the warm-bath and the tepid-bath, and all other baths, if there are any.

In treating of the Passions, he gives an ingenious dissertation on amulets or charms, which he thinks act more on the imagination than on the body. Love, he says, "is a *cordial drop*, which Heaven has thrown into the bitter cup of life," (we presume, in order to prevent its griping.) This is prettily expressed: at the same time, he approves of love only after matrimony, which is highly proper. Sorrow, we are informed, "strikes the heart and makes it flutter;" and that it produces flatulency; and that, in spite of scepticism, it is perfectly true that people do die of broken hearts.

The diseases of literary men are next considered; and the author thinks that, although diseases do not come in at the doors or windows of their studies, they are liable to the misfortune, nevertheless, of having them generated in their own bodies. Tired brains, he says, cannot sleep: and he quotes Latin to prove it, and therefore it must be true. Torpid bowels, Butler, Sterne, Professor Porson, digestive organs, and acidities in the stomach, are next mentioned, and discussed clerkly. Sleep is said to be a mysterious state, and that "the night-consumer lies like Polyphemus immersed in the fumes of debauch." Two notes of admiration follow this sentence; and then, after another note of admiration, he takes occasion to observe, that "the valetudinarian will ponder on these things." He then treats of the night-mare and Walter Scott; Cowper; sedentary employments; chesnuts; and sour wine. He threatens, in a note (p. 210), to publish another book; and observes, in another note, that those who are much fatigued before dinner may sleep a little after dinner.

On the score of medicine, the author seems to think highly of "purgatives, diaphoretics, silagogues, cholagogues, &c." which means, we suppose, all other "gogues." He thinks blue-pill and aloes better than balm of Gilead or Dutch drops; and recommends bleeding in the spring and fall, the benefits of which are confirmed by a note taken from the Dublin Hospital Reports. The author then announces that the volume is finished.

It is usual, on having completed the analysis of a work, to give some sort of peroration or summing-up of evidence, in which the merits of the author and his book are exhibited quaintly, and for the convenience of the memory, in a portable shape. The practice of peroration is also a good one *for effect;* it is like a good chorus, or a great blaze of light, at the termination of a play, when the curtain drops. Now, although we do not altogether disclaim this precedent, yet we do not hold ourselves so strictly bound by it, but that we may deviate from the custom if we please. It is, however, impossible to dismiss this volume without expressing in a few words our opinion of the book; and without giving, at the same time, a brief admonition to the author. With respect to the book, then, it appears to be a thing made in part out of the little trumperies of a life ill-spent for creditable authorship; but *chiefly* composed of shreds and patches, taken out of an Encyclopedia, or such work, and a few modern authorities, and these not always acknowledged. These materials are clumsily put together, and amalgamated by a headlong fury of writing, which joins things together that nature has endeavoured to keep asunder; runs incongruities, monstrosities, and absurdities, into the same sentence; and, by an abuse of terms, gives to those sentences an air of pregnant meaning, which amounts, in fact, only to the most unintelligible nonsense. We should not take the trouble to say so much of *this book*, for in truth the volume is not worth the value of the paper which we have consumed in these remarks; but there are many books of the same kind; books which profess to teach, while they only mislead; books which pretend to enlighten, and serve only to confuse; books which assume the air of science, while they exhibit nothing but ignorance; books which pretend to extend knowledge, while they only confine it; books which assume the direction of public taste, whilst they serve merely to corrupt it; books which pretend to be the only catholic and canonical books, but which are conceived in ignorance and born of stupidity; books which profess to be *practical*, and contain nothing but the worst, because the most ill-constructed, theory: there are such books; and, as the writers of them have got the ear of such of the public as have no time for nice discrimination, they pass for current coin, to the exclusion of such as are intrinsically valuable; which, being formed by superior workmen, are perhaps neglected in the age which produced them, and admired or wondered at in the age which succeeds to it.

The second part of our peroration, to wit, our admonition to the author, is soon made. We have very little more to say to him, than that which his vanity will not allow him to believe, namely, that his talents are not of that order which will qualify him even for a writer of mediocrity: we therefore recommend him in future to spare his ink. To this our individual opinion he may perhaps oppose the argument, "the public thinks differently, witness this *third* edition." The argument is very forcible: at the same time our author may be reminded of the great sale of "Solomon's Guide to Health," which, together with such works as our author's, the booksellers get rid of rapidly, because, like Peter Pindar's razors, "they are made for sale."

