# Errata

**Published:** 1820-07

**Authors:** 


ERRATA.

Page 305, last line, for *process* read *prostate*.

