# Corrections to Be Made in the Last Number

**Published:** 1820-11

**Authors:** 


*** *Corrections to be made in the last Number.*—Page 326, line 29, add a *comma* after *remarks;* and, in the same page, line 39, omit the inverted commas: the passage they include is by mistake given as a precise quotation, though the sum and substance of it is stated in the 73d page of the work referred to in it.

